# Supplementary material for: Mechanisms governing the pioneering and redistribution capabilities of the non-classical pioneer PU.1
Source: Nat Commun. 2020 Jan 21;11:402. doi: 10.1038/s41467-019-13960-2 (PMC6972792; doi:10.1038/s41467-019-13960-2)
Supplement: Supplementary file 7 — Source data [file 41467_2019_13960_MOESM7_ESM.zip › Source_Data/FigureS5/FigureS5B_MotifsETS1specificPeaks/homerResults/motif16.similar.html]

motif16

## Information for motif16

T
A
G
C
A
T
C
G
A
G
T
C
C
T
A
G
T
A
G
C
A
C
T
G
A
C
T
G
A
G
T
C
T
C
G
A
A
G
T
C
A
C
T
G
A
T
G
C
  
Reverse Opposite:  

T
A
C
G
T
G
A
C
T
C
A
G
A
G
C
T
T
A
C
G
A
G
T
C
T
G
A
C
A
T
C
G
A
G
T
C
T
C
A
G
T
A
G
C
A
T
C
G
  

|  |  |
| --- | --- |
| p-value: | 1e-22 |
| log p-value: | -5.239e+01 |
| Information Content per bp: | 1.634 |
| Number of Target Sequences with motif | 131.0 |
| Percentage of Target Sequences with motif | 11.54% |
| Number of Background Sequences with motif | 315.4 |
| Percentage of Background Sequences with motif | 3.86% |
| Average Position of motif in Targets | 99.2 +/- 45.1bp |
| Average Position of motif in Background | 102.7 +/- 83.5bp |
| Strand Bias (log2 ratio + to - strand density) | -0.2 |
| Multiplicity (# of sites on avg that occur together) | 1.16 |
| Motif File: | file (matrix) reverse opposite |

### Similar de novo motifs found

|  |  |  |  |  |  |  |  |
| --- | --- | --- | --- | --- | --- | --- | --- |
| Rank | Match Score | Redundant Motif | P-value | log P-value | % of Targets | % of Background | Motif file |
| 1 | 0.763 | A T C G A G T C A T C G G A T C C T A G A T G C C A G T A T C G A T G C G A T C A G C T A C T G T A G C | 1e-18 | -43.654510 | 5.55% | 1.12% | motif file (matrix) |
